# 

**Published:** 1881-07

**Authors:** 


### Table of Contents.

### REPORTS OF SOCIETIES.

The American Medical Association ........................ 193

### SELECTIONS.

Some Observations on the Carlsbad Treatment ........................ 220
On the Pathology and Treatment of Chorea ........................ 226
Ulcerative Endocarditis ........................ 234
Angina Pectoris ........................ 241
Shall Physicians Dispense their own Medicine? ........................ 242

### EDITORIAL.

Scarlet Fever ........................ 246

### BIBLIOGRAPHICAL.

Supplement to Ziemssen's Cyclopedia of the Practice of Medicine... 247

### EXCERPTA.

Don't Bathe After Meals ........................ 248
Dyspepsia Caused by Tight Lacing ........................ 249
Danger in Fruit Cans ........................ 249
Conjunctivitis: Is Astringent Treatment Called for? ........................ 249
Quarantine Conundrums ........................ 251
Oleomargarine Again ........................ 252
The Opinion as to Quinine in Pneumonia ........................ 252
Treatment of Diphtheria ........................ 253
On the Absorption and Elimination of Quinine ........................ 254
Salicylic Acid as an Adulteration ........................ 254
Diploma Vending ........................ 255
Gurjun Oil in Leprosy ........................ 255
Death from Opening an Abscess ........................ 256
Local Application for the Relief of Erysipelas ........................ 256
Syringing the Ear ........................ 256

